# Angiographic study of the transdural collaterals at the anterior cranial fossa in patients with Moyamoya disease

**DOI:** 10.7150/ijms.48308

**Published:** 2020-07-25

**Authors:** Kun Hou, Guichen Li, Yunbao Guo, Baofeng Xu, Kan Xu, Jinlu Yu

**Affiliations:** 1 Department of Neurosurgery, The First Hospital of Jilin University, Changchun, 130021, China.; 2 Department of Neurology, The First Hospital of Jilin University, Changchun, 130021, China.

**Keywords:** Moyamoya disease, Anterior cranial fossa, Transdural collateral, Anterior ethmoid artery, Posterior ethmoid artery

## Abstract

Unlike its parietal, temporal, and occipital counterparts, the frontal lobe has a broad basal surface directly facing the anterior cranial fossa dura mater which could permit establishment of transdural collaterals (TDCs) with the frontal lobe. Studies on the TDCs from the anterior cranial fossa in moyamoya disease (MMD) are scarce and inadequately investigated. A retrospective study of 100 hemispheres in 50 patients who were diagnosed with MMD by catheter angiography between January 2015 and June 2019 was performed in our institution. TDCs through the anterior ethmoid artery (AEA) or posterior ethmoid artery (PEA) were divided into 3 types respectively based on their respective angioarchitecture. Furthermore, we also studied TDCs to the temporal, parietal, and occipital lobes and collaterals from the posterior circulation to the territory of the anterior cerebral artery. TDCs through the AEA and PEA were identified in 89 (89/100, 89%) and 73 (73/100, 73%) of the hemispheres. The vascularization state of the frontal lobe was good in 89 (89/100, 89%) hemispheres. Rete mirabile and TDCs through the PEA were statistically different among patients with different Suzuki stages. No statistical difference was noted in TDCs through the AEA, frontal TDCs from other sources, and the vascularization state of the frontal lobe with regard to different Suzuki stages. TDCs through the AEA and PEA at the anterior cranial fossa play a very important role in compensating the ischemic frontal lobe. The frontal lobe could be well compensated in most of the patients with TDCs at the anterior cranial fossa.

## Introduction

Moyamoya disease (MMD) is characterized by progressive steno-occlusive alteration of the supraclinoid portion of the internal carotid artery (ICA) and the beginning of its two main branches. During the irreversible progression of disease, as a compensatory mechanism, puff-like collaterals to the ischemic cerebral parenchyma form at the base of the brain 1.

When MMD occurs, the frontal and parietal lobes are most commonly and severely involved 2. And, as a compensating mechanism, the external carotid artery and vertebrobasilar system would provide collaterals to the ischemic frontal and parietal cortex 3-5. Unlike its parietal counterpart, the frontal lobe has a broad basal surface directly facing the anterior cranial fossa dura mater which could form collateral circulation with the frontal lobe. These collaterals always penetrate the dura mater to anastomose with the brain pial arteries, hence are called transdural collaterals (TDCs) 1.

However, to present a detailed prospect of the TDCs at the anterior cranial fossa, we performed a retrospective study of the patients with MMD in our institution. Besides, TDCs from other sites to the frontal, parietal, temporal, and occipital lobes and collaterals from the posterior circulation to the frontal lobe were also investigated.

## Materials and Methods

A retrospective study of the patients between January 2015 and June 2019 was performed in our institution. This study was approved by the institutional ethics committee (approval number: 2020-084). The diagnosis of MMD and the evaluation of collaterals were conducted by two experienced neurointerventionists (Kun Hou and Jinlu Yu). In case of disagreement between the two evaluators, a third neurointerventionist (Kan Xu) would be consulted. The Suzuki stage of the studied patients would also be provided 6.

### Inclusion criteria

- No evident encephalomalacia was identified in the frontal lobe (lacunar cerebral infarction is permitted);
- No history of previous craniotomy or craniectomy;
- A 6-vessel angiography with anteroposterior and lateral views must be performed;
- Diagnosed with typical bilateral MMD;
- TDC must be identified in bilateral anterior skull base.

### TDCs from the anterior cranial fossa

The ophthalmic artery (OphA) gives rise to the anterior ethmoid artery (AEA) and the posterior ethmoid artery (PEA). The AEA and PEA are the primary source of TDCs to the frontal lobe at the anterior cranial fossa 7, 8. In addition, the middle meningeal artery (MMA), internal maxillary artery (IMA), and facial artery (FA) could also provide TDCs to the frontal lobe at the anterior cranial fossa through the AEA and PEA. Rete vasculosum (or rete mirabile) might also be formed at the anterior cranial fossa 1.

#### TDCs through the AEA

**Figure 1:**
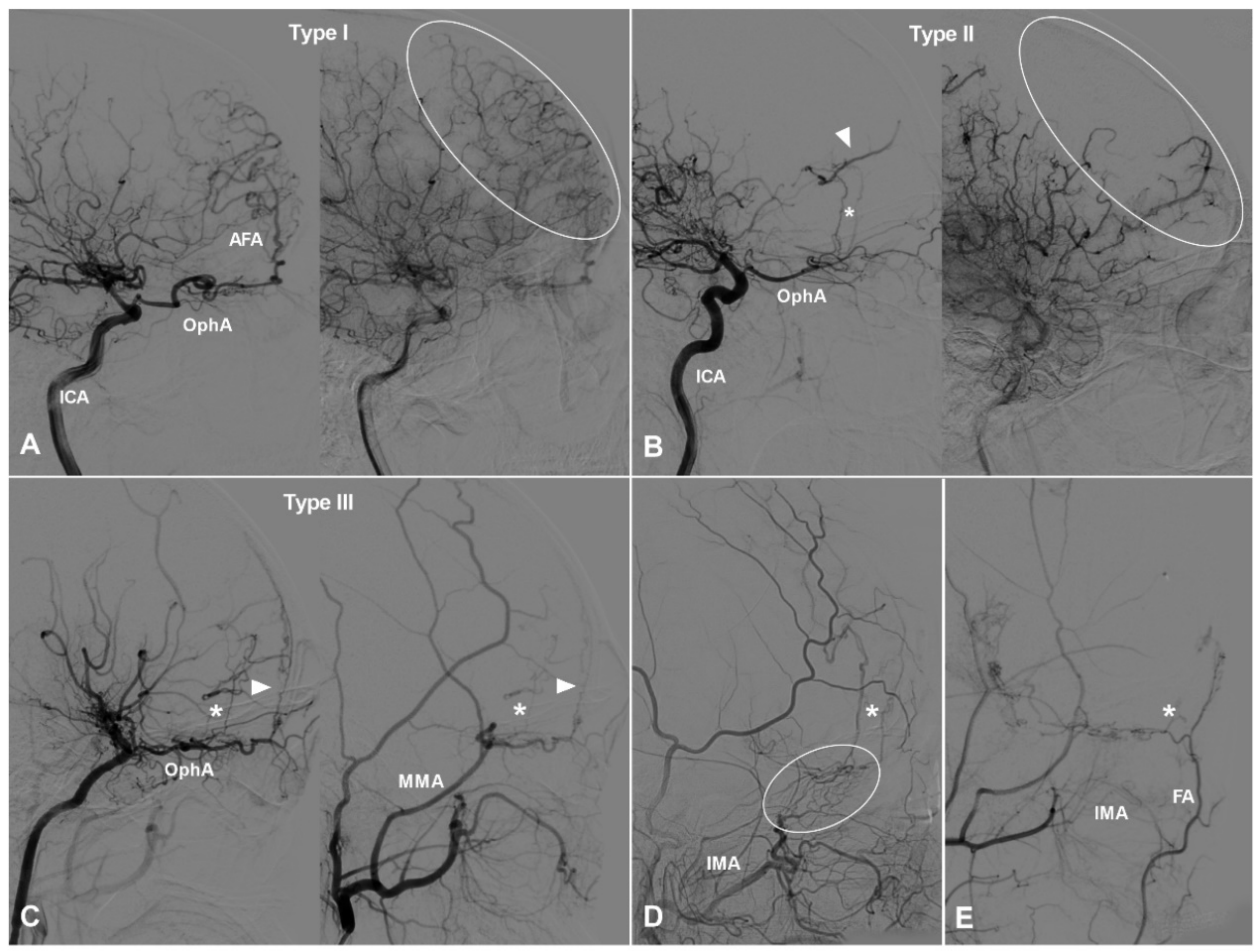
** TDCs through the anterior ethmoid artery.** A, Angiogram of the ICA (left) in lateral view shows the AEA courses anteriorly after originating from the OphA, then turns superiorly and anastomoses with the AFA and the cortical artery of the frontal lobe (type I). The same angiogram (right) in late arterial phase shows the frontal lobe (oval) is well vascularized by collaterals. B, Angiogram of the ICA (left) in lateral view shows the AEA (asterisk) courses superiorly after originating from the OphA and anastomoses directly with the cortical artery (arrow head) of the frontal lobe (type II). The same angiogram (right) in late arterial phase shows the frontal lobe (oval) is poorly vascularized by collaterals. C, Angiogram of the ICA (left) in lateral view shows the AEA courses superiorly (asterisk) and anteriorly and anastomoses with the cortical artery and AFA (arrow head) (type III). Angiogram of the ECA (right) in the same patient shows MMA also participates in the TDCs through the AEA (asterisk and arrow head). D, Angiogram of the ECA shows the IMA (oval) also participates in the TDCs through the AEA (asterisk). E, Angiogram of the ECA shows the FA (oval) also participates in the TDCs through the AEA (asterisk). **Abbreviations:** AEA, anterior ethmoid artery; AFA, anterior falx artery; ECA, external carotid artery; FA, facial artery; ICA, internal carotid artery; IMA, internal maxillary artery; MMA, middle meningeal artery; OphA, ophthalmic artery; TDC, transdural collateral.

TDCs through the AEA can be divided into 3 types. Type Ⅰ: The AEA courses anteriorly and continues as the anterior falx artery (AFA). The AFA courses superiorly and posteriorly and anastomoses with the cortical arteries of the frontal lobe 9. Type Ⅱ: The AEA courses superiorly and anastomoses directly with the cortical arteries of the frontal lobe. Type Ⅲ: The AEA gives rise to multiple branches coursing anteriorly and superiorly and anastomoses with the cortical arteries of the frontal lobe. TDCs from the AEA are illustrated in Figure 1.

#### TDCs through the PEA

**Figure 2:**
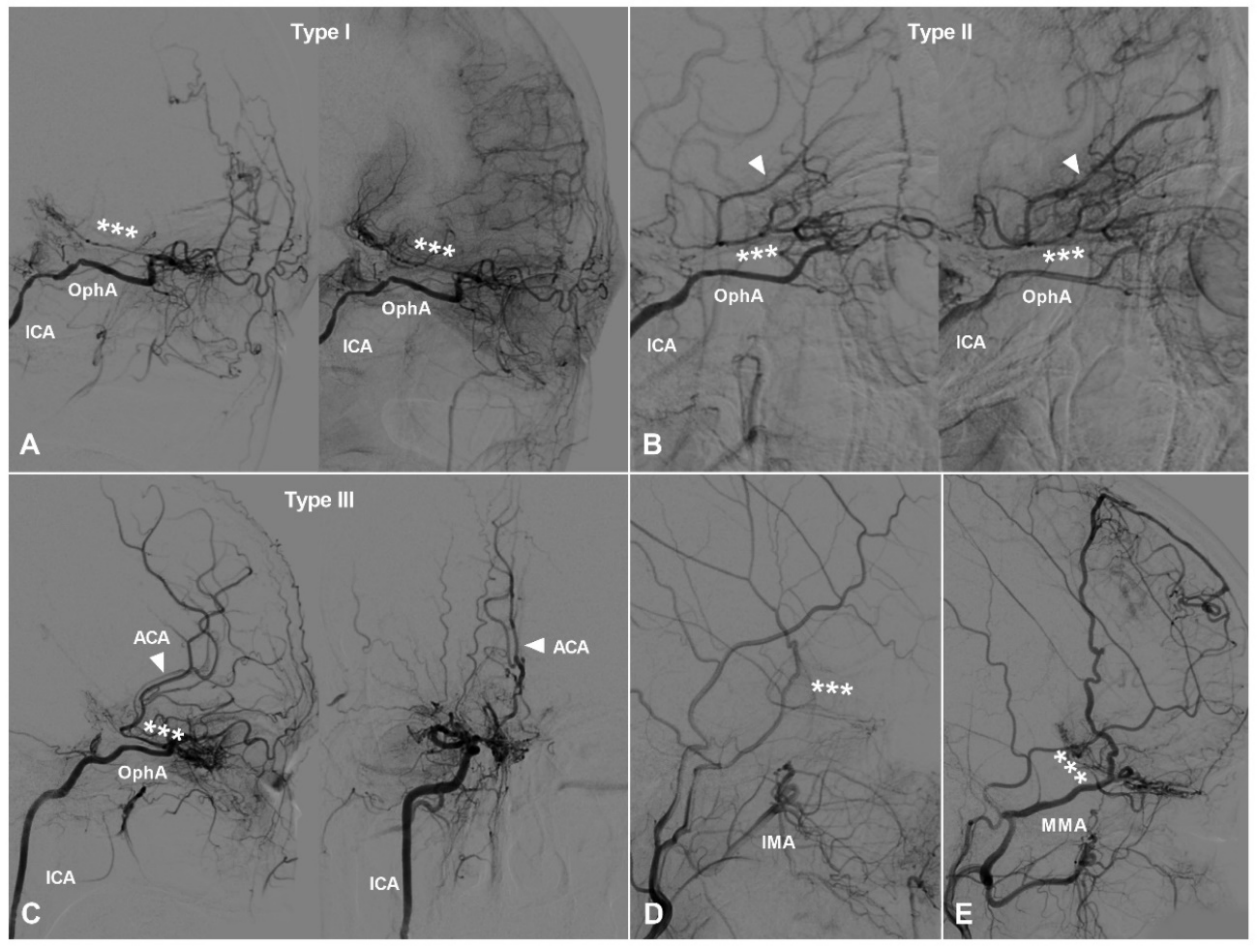
** TDCs through the posterior ethmoid artery.** A, Angiogram of the ICA in lateral view shows the PEA (asterisks) courses posteriorly after originating from the OphA and anastomoses with the orbitofrontal artery at the frontal base (type I). B, Angiogram of the ICA in lateral view shows the PEA (asterisks) courses posteriorly after originating from the OphA and anastomoses with the branches of the ACA (type II). C, Angiogram of the ICA in lateral (left) and AP (right) views shows the PEA (asterisks) courses posteriorly after originating from the OphA and anastomoses with the main trunk (arrow head) of the ACA (type III). D, Angiogram of the ECA shows the IMA also participates in the TDC of PEA (asterisks). E, Angiogram of the ECA shows the MMA also participates in the TDC of PEA (asterisks). **Abbreviations:** ACA, anterior cerebral artery; AP, anteroposterior; ECA, external carotid artery; ICA, internal carotid artery; IMA, internal maxillary artery; MMA, middle meningeal artery; OphA, ophthalmic artery; TDC, transdural collateral.

TDCs through the PEA can also be divided into 3 types. Type Ⅰ: The PEA courses posteriorly and only anastomoses with the orbitofrontal artery at the frontal base. Type Ⅱ: The PEA courses posteriorly and anastomoses with the other big branches of the anterior cerebral artery (ACA). Type Ⅲ: The PEA courses posteriorly and anastomoses with the main trunk of ACA. TDCs from the PEA are illustrated in Figure 2.

### TDCs to the other surfaces of the frontal lobe and the other lobes

**Figure 3:**
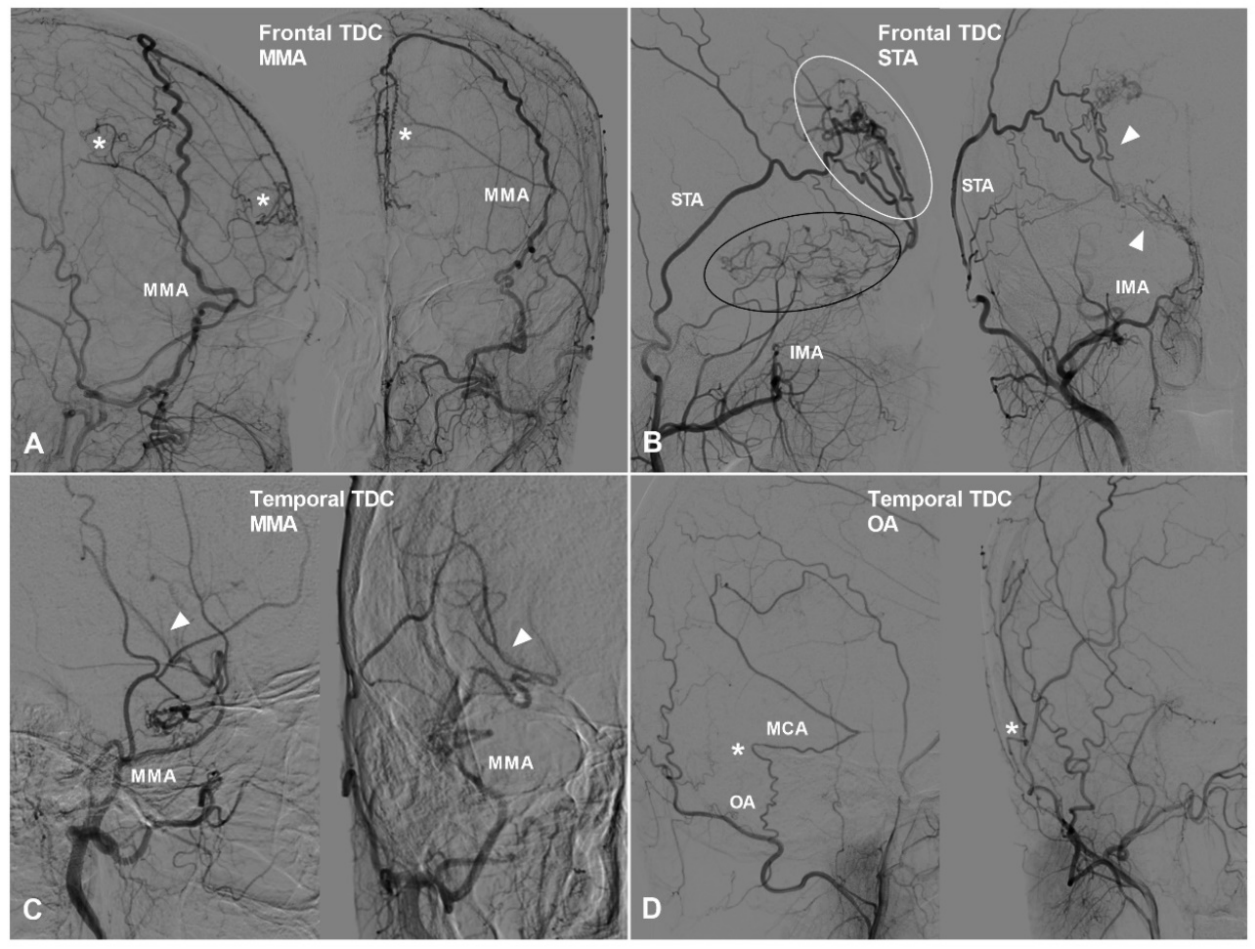
** The frontal and temporal TDCs.** A, Angiogram of the ECA in lateral (left) and AP (right) views shows the MMA provides TDC by anastomosing with the midline arteries (asterisk). B, Angiogram of the ECA in lateral (left) and AP (right) views shows the STA (white oval) and IMA (black oval) provide TDCs to the brain. Besides, the STA and IMA also form anastomosis (arrow head). C, Angiogram of the ECA in lateral (left) and AP (right) views shows TDC from the MMA anastomoses with the MCA (arrow head). D, Angiogram of the ECA in lateral (left) and AP (right) views shows TDC from the OA anastomoses with the MCA (asterisk). **Abbreviations:** ECA, external carotid artery; IMA, internal maxillary artery; MMA, middle meningeal artery; OA, occipital artery; TDC, transdural collateral.

**Figure 4:**
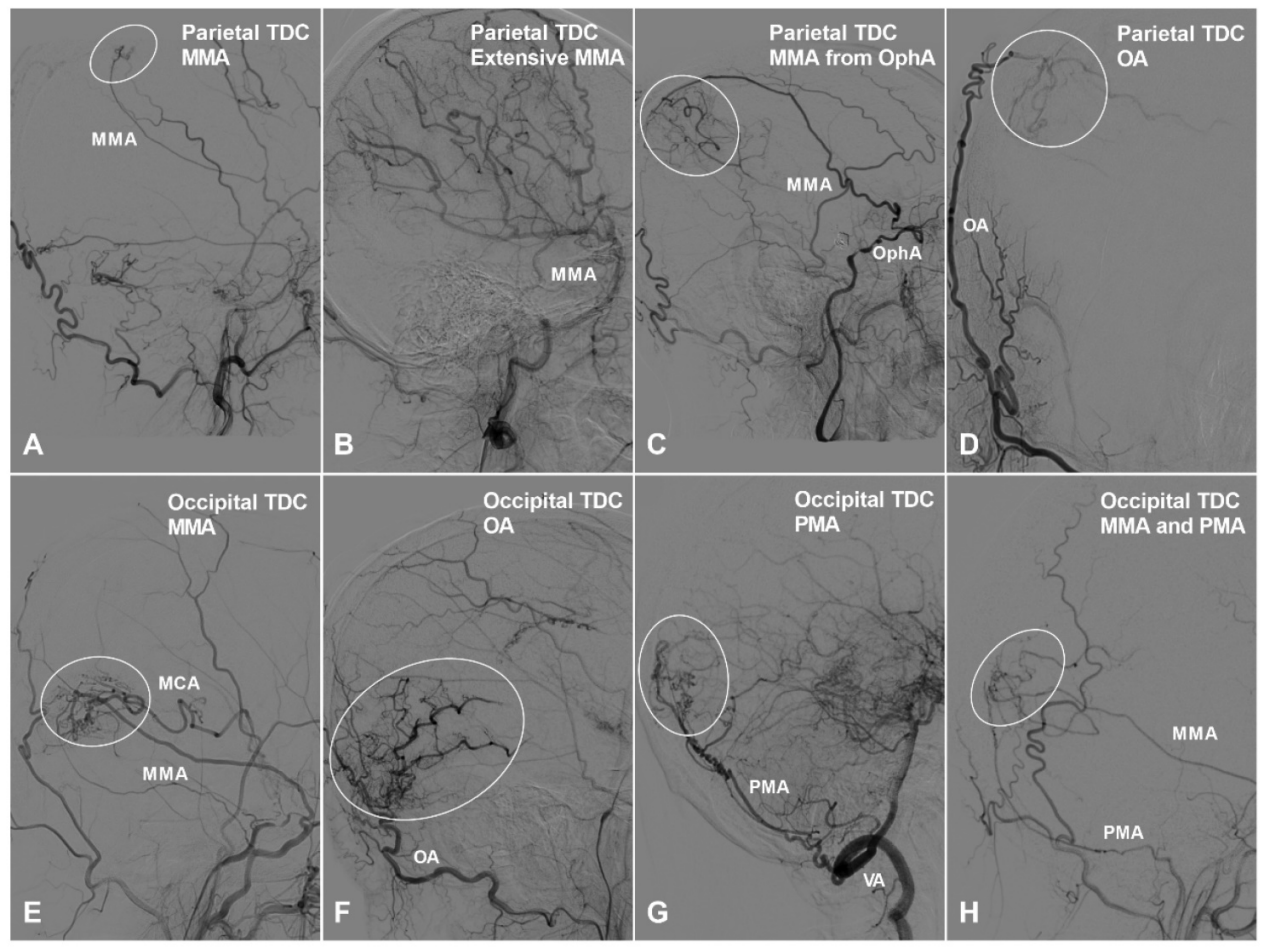
** The parietal and occipital TDCs.** A, Angiogram of the ECA in lateral view shows a small parietal TDC originates from the MMA (oval). B, Angiogram of the ECA in lateral view shows the MMA provides extensive TDCs to the parietal cortex. C, Angiogram of the ECA in lateral view shows the MMA originating from the OphA provides TDC (oval) to the parietal cortex. D, Angiogram of the ECA in anteroposterior view shows the OA provide TDC (circle) to the parietal cortex. E, Angiogram of the ECA in lateral view shows TDC (oval) from the MMA anastomoses with the MCA at the occipital cortex. F, Angiogram of the ECA in lateral view shows TDC (oval) from the OA anastomoses with the occipital cortical arteries. G, Angiogram of the VA in lateral view shows TDC (oval) from the PMA anastomoses with the occipital cortical arteries. H, Angiogram of the ECA in lateral view shows TDCs (oval) from the PMA and MMA anastomose with the occipital cortical arteries. **Abbreviations:** ECA, external carotid artery; MCA, middle cerebral artery; MMA, middle meningeal artery; OA, occipital artery; OphA, ophthalmic artery; PMA, posterior meningeal artery; TDC, transdural collateral; VA, vertebral artery.

Apart from TDCs at the anterior cranial fossa, the frontal lobe can also receive collateral circulation at its lateral, superior, and medial surfaces. The MMA, superficial temporal artery (STA), and FA could pierce the dura mater and anastomose with the cortical arteries of the frontal lobe. Furthermore, we have also studied TDCs to the temporal, parietal, and occipital lobes. TDCs from the MMA, STA, and FA are illustrated in Figure 3 and 4.

### Collaterals from the posterior circulation to the frontal lobe

**Figure 5:**
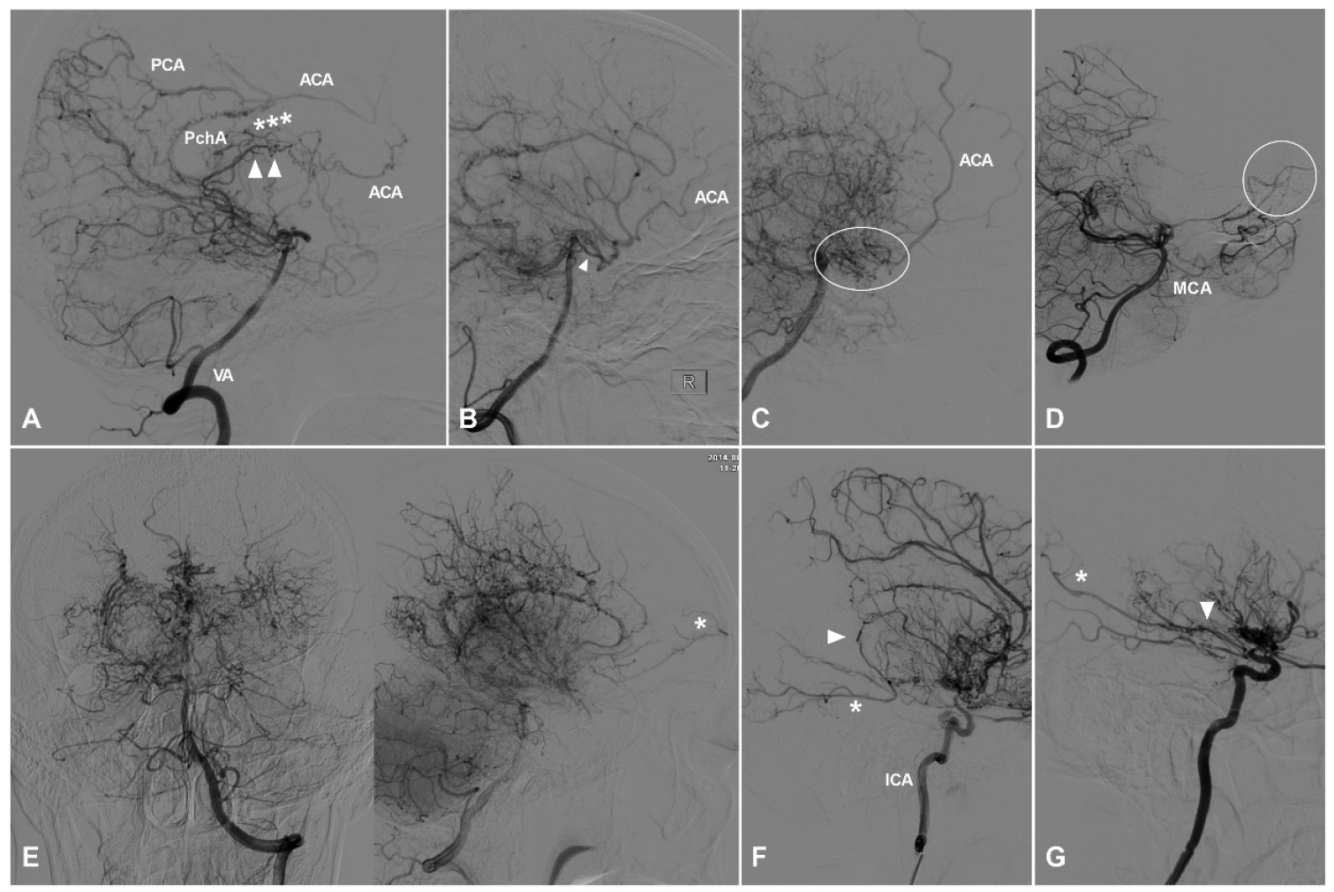
** Collaterals from the posterior circulation.** A, Angiogram of the VA in lateral view shows the PCA and PChA anastomose with the posterior portion of the ACA (asterisks), forming a callosal circle. In addition, ventricular collaterals (arrow heads) could also be noted in this patient. B, Angiogram of the VA in lateral view shows the posterior circulation provides blood supply to the territory of ACA via the PComA (arrow head). C, Angiogram of the VA in lateral view shows the posterior circulation provides blood supply to the territory of ACA via the moyamoya-like vessels (oval) at the cranial base. D, Angiogram of the VA in lateral view shows the posterior circulation provides blood supply to the territory of ACA (oval) via pial branches of the MCA, namely the PCA-MCA-ACA pathway. E, Angiogram of the VA in AP (left) and lateral (right) views shows moyamoya-like alteration in the posterior circulation. The posterior circulation could still provide collateral (asterisk) to the anterior circulation. F, Angiogram of the ICA in lateral view shows the PChA (arrow head) anastomoses with the PCA (asterisk) via retrograde blood supply. F, Angiogram of the ICA in lateral view shows the AChA (arrow head) anastomoses with the PCA (asterisk) via antegrade blood supply. **Abbreviations:** ACA, anterior cerebral artery; AChA, anterior choroidal artery; AP, anteroposterior; ICA, internal carotid artery; MCA, middle cerebral artery; PCA, posterior cerebral artery; PChA, posterior choroidal artery; PComA, posterior communicating artery; VA, vertebral artery.

Most of the time, arteries in the posterior circulation are not involved in patients with MMD and can provide compensating collaterals to the anterior circulation 4. These collaterals mainly comprise of callosal circle, ventricular collaterals, and cranial base collaterals 1. These collaterals are illustrated in Figure 5. As the posterior communicating artery (PComA) and anterior choroidal artery (AChA) could also participate in the posterior to anterior compensation 10, 11, enlargement of the the PComA and AChA was also evaluated in this study.

#### Callosal circle

The posterior choroidal artery (PChA) and/or cortical branches of the posterior cerebral artery (PCA) could anastomose with the ACA at the posterior part of the corpus callosum and perfuse retrogradely to the territory of ACA, which is called callosal circle 12.

#### Ventricular collaterals

In rare circumstance, the posterior circulation could provide blood supply to the frontal lobe via the ventricular arterial network, which is called ventricular collaterals 13.

#### Cranial base collaterals

The posterior circulation could provide blood supply to the territory of ACA via anastomosing with bridging vessels at the cranial base. These bridging cranial base vessels mainly include the moyamoya-like network, the remnant PComA, and the cortical branches of middle cerebral artery (MCA) 14. The complete collateral pathways are from PCA-MCA to ACA, from PCA-PComA to ACA, and from moyamoya-like network to ACA.

### Vascularization state of the frontal lobe

The vascularization state of the frontal lobe was evaluated by contrast staining in late arterial and venous phases during catheter angiography. The frontal lobe might receive compensating collaterals at the anterior cranial fossa, from the posterior circulation, and from other sources. If no blank area of the frontal lobe was noticed during angiography, the frontal lobe was considered with good vascularization. Otherwise, it was considered with poor vascularization.

### Statistical analysis

Statistical assessment was performed using SPSS 25.0 (IBM Corp., Armonk, NY, USA). Chi-square test or Fisher exact test was used to analyze count data. *P<*0.05 was considered with statistical difference.

## Results

### General information

**Figure 6:**
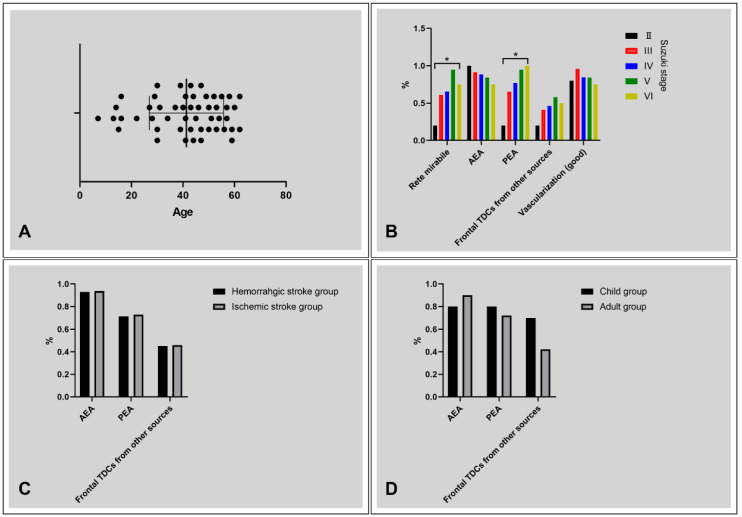
** Diagrams of statistical analysis.** A, Age distribution of the patients, ranging from 7 to 62 years. B, The rate of rete mirabile, AEA TDCs, PEA TDCs, frontal TDCs from other sources, and vascularization with regard to Suzuki stage. The rate of rete mirabile and PEA TDCs increases with the progression of Suzuki stage (P < 0.05). C, No statistical difference was noted between the patients with ischemic and hemorrhagic clinical presentation with regard to frontal TDCs. D, No statistical difference was noted between the child and adult groups with regard to frontal TDCs. * P < 0.05. **Abbreviations:** AEA, anterior ethmoid artery; PEA, posterior ethmoid artery; TDC, transdural collateral.

Fifty (21 males, 42%) consecutive patients, aging from 7-62 (41.32 ± 14.42) years, were identified. Five (5/50, 10%) children patients (<18 years) were identified. The age distribution of the patients was presented in Figure 6A. Twenty-four (24/50, 48%) patients had ischemic presentation, 21 (21/50, 42%) patients had hemorrhagic presentation. Five (5/50, 10%) patients presented with other complaints. The Suzuki stage were II, III, IV, V, and VI in 5 (5/100, 5%), 46 (46/100, 46%), 26 (56/100, 26%), 19 (19/100, 19%), and 4 (4/100, 4%) hemispheres.

### TDCs through the AEA

Presence of TDCs through the AEA was identified in 89 (89/100, 89%) hemispheres. Type I TDCs through the AEA were identified in 44 (44/100, 44%) hemispheres, of which 36 were from the OphA, 6 were from the OphA and IMA, 1 was from the OphA, IMA, and FA, and 1 was from the MMA. Type II TDCs were identified in 9 (9/100, 9%) hemispheres, all of which were from the OphA. Type III TDCs were identified in 36 (36, 36/100, 36%) hemispheres, of which 31 were from the OphA and 5 were from the OphA and IMA.

### TDCs through the PEA

Presence of TDCs through the PEA was identified in 73 (73/100, 73%) hemispheres. Type I TDCs through the PEA were identified in 45 (45/100, 45%) hemispheres, of which 36 were from the OphA, 7 were from the OphA and IMA, 1 was from the OphA, IMA, and FA, and 1 was from the MMA. Type II TDCs were identified in 12 (12/100, 12%) hemispheres, all of which were from the OphA. Type III TDCs were identified in 16 (16/100, 16%) hemispheres, of which 14 were from the OphA and 2 were from the OphA and IMA.

### TDCs to the other sites

#### TDCs to the other surfaces of frontal lobe

TDCs to the other surfaces of frontal lobe were identified in 45 (45/100, 45%) hemispheres, of which 39 were from the MMA, 5 from the STA, and 1 from the FA.

#### TDCs to the other lobes

TDCs to the temporal, parietal, and occipital lobes were identified in 36 (36/100, 36%) hemispheres, of which 1 TDC was identified in 25 hemispheres, 2 TDCs were identified in 6 hemispheres, 3 TDCs were identified in 2 hemispheres, 4 TDCs were identified in 1 hemisphere, and extensive TDCs were identified in 2 hemispheres.

### Collaterals from the posterior circulation

#### Callosal circle

Presence of callosal circle was identified in 40 (40/50, 80%) patients. Callosal circle from the PCA was identified in 6 (6/50, 12%) patients, from the PChA in 18 (18/50, 36%) patients, and from the PCA and PChA in 16 (16/50, 32%) patients.

#### Ventricular collaterals

Ventricular collaterals were identified in 7 (7/50, 14%) patients.

#### Cranial base collaterals

Cranial base collaterals were present in 31 (31/50, 62%) patients. Cranial base collaterals from the PCA-MCA to the ACA were identified in 14 (14/50, 28%) patients. From the PCA-PComA to the ACA was identified in 12 (12/50, 24%) patients. From the combined PCA-MCA and PCA-PComA to the ACA was identified in 1 (1/50, 2%) patient. From the moyamoya-like network to the ACA were identified in 3 (3/50, 6%) patients. Besides, 1 (1/50, 2%) patient was identified to be from the combined moyamoya-like network and PCA-MCA to the ACA.

#### Contribution of the enlarged PComA and AChA

The enlarged PComA and AChA could provide blood supply to the posterior circulation and hence increase the blood flow from collaterals to the ACA territory. Enlarged PComA and AChA were identified in 56 (56/100, 56%) and 17 (17/100, 17%) hemispheres respectively. Besides, the PCA was noted to originate from the AChA and PChA in 6 (6/100, 6%) and 3 (3/100, 3%) hemispheres, respectively.

### Posterior MMD

Posterior MMD was identified in 3 (3/50, 6%) patients.

### Vascularization state of the frontal lobe

The vascularization state of the frontal lobe was good in 89 (89/100, 89%) hemispheres and poor in 11 (11/100, 11%) hemispheres.

### Statistical analysis

#### Collaterals and vascularization state of the frontal lobe with regard to Suzuki stages

The Suzuki stage, collaterals, and vascularization state of the frontal lobe were summarized in Table 1. Rete mirabile and TDCs through PEA were statistically different among patients with different Suzuki stages (Table 2). No statistical difference was noted in TDCs through the AEA, frontal TDCs from other sources, and the vascularization state of the frontal lobe among different Suzuki stages (Figure 6B).

#### Frontal TDCs with regard to clinical manifestations

No statistical difference was noted in TDCs through the AEA, PEA, and Frontal TDCs from other sources between the ischemic and hemorrhagic groups (Table 3, Figure 6C).

#### Frontal TDCs with regard to different age groups

No statistical difference was noted in TDCs through the AEA, PEA, and Frontal TDCs from other sources between the children and adult groups (Table 4, Figure 6D).

## Discussion

MMD is an uncommon cerebrovascular disease characterized by progressive stenosis of the terminal portion of the internal carotid artery and its main branches, which is more popular in East Asian countries 15, 16. Due to the ischemic state in anterior circulation, collateral circulation always forms in patients with MMD 17. According to their origin, the collaterals of MMD can be classified as moyamoya vessels, leptomeningeal anastomosis from the posterior circulation, and TDCs 18. In MMD, moyamoya-like vessels mainly locate at the basal ganglia and thalamus, anterior cranial fossa, and cerebral convexity 19. Besides, periventricular collaterals can also form 13, 20.

Currently, few studies evaluate the collaterals of MMD according to their specific locations. The frontal lobe is a peculiar area and surrounded by arterialized dura mater at its basal, lateral, superior, and medial surfaces. The arteries supplying the dura mater can provide collaterals to the frontal lobe. Besides, the posterior circulation could also provide collaterals to the frontal lobe 14. Among the aforementioned collaterals, TDCs from the anterior cranial fossa are very important but not yet well studied.

In order to further explore the TDCs at the anterior cranial fossa in patients with MMD, we conducted this study. As large areas of hemorrhage or ischemia might damage the neighboring collaterals, the included patients in the current study must have morphologically intact frontal lobe or no evident frontal encephalomalacia secondary to ischemia or hemorrhage could be noticed. Besides, patients with only unilateral anterior cranial fossa TDCs were also excluded. Because we also investigated the TDCs from other sites, which might be different in the cerebral hemispheres with and without anterior cranial fossa TDCs.

The patients included in this study are those with bilateral TDCs from the anterior cranial fossa. By analyzing the 50 patients, we noted the collaterals to the frontal lobe were from the following sources: TDCs from the anterior cranial fossa, TDCs from the lateral surface of the frontal lobe, TDCs from medial surface of the frontal lobe, and collaterals from the posterior circulation. These collaterals conjointly guarantee the vascularization of the frontal lobe 21.

The frontal lobe has a relative broad basal surface. Hence, the AEA and PEA could form collateral circulation with the cortical branches at the frontal base 22. At the embryonal stage, the orbit is supplied by the ventral and dorsal OphAs originated from the ACA and internal carotid artery (ICA) siphon, respectively. Later, the dorsal OphA regresses. The ventral OphA anastomoses with the ICA near the anterior clinoid process and its proximal segment regresses too 23. In rare circumstance, the persistent ventral OphA can be preserved, which connects the OphA and ACA 24. In our study, type III TDC through the PEA was similar with the persistent ventral OphA. But it is difficult to distinguish between them. The reported cases of persistent ventral OphA are congenital but not postnatal anomaly 24. In the case of MMD, compensating collaterals would form. Hence, the type III PEA TDC is unlikely to be persistent primitive ventral OphA.

Though the collateral circulation at the anterior cranial fossa has been reported previously, no detailed study has been performed 1. In the present study, we classified the TDCs at the anterior cranial fossa according to the characteristics of AEA and/or PEA involvement, which we believe to be very important.

In this study, we found that TDCs through the AEA accounted for 89% of the hemispheres in patients with anterior cranial fossa TDCs. Though the rate of TDCs through the AEA seems decreasing with the progression of Suzuki stage, no statistical difference was noted. This might imply that the AEA TDCs are easier to establish and can be formed at the early stage of MMD, which accompanies with the whole process of MMD. However, TDCs through the PEA accounted for 73% of the hemispheres in patients with anterior cranial fossa TDCs. Statistical analysis showed that with the progression of Suzuki stage, the rate of PEA TDCs increases. TDC through the PEA must anastomose with the branches or the main trunk of ACA, which is more difficult to form. This scenario has some similarity with arteriovenous fistula in the anterior cranial fossa, of which the AEA is more commonly involved as the feeding artery than the PEA 25. This might denote that PEA TDCs are uncommon and gradually form with the progression MMD.

Generally speaking, TDCs through the AEA and PEA mainly originate from the OphA. They can also originate from the MMA, IMA, and FA. These TDCs could anastomose directly with the AEA and PEA, or by forming rete mirabile. In this study, we found that the incidence of rete mirabile positively correlated with the Suzuki stage, which indicated that the rete mirabile might also gradually form with the progression of MMD.

The TDCs to the other surfaces of the frontal lobe were also investigated. We found that, in rare circumstances, the STA and FA can penetrate the cranium and dura mater and anastomose with the cortical branches of the lateral surface of the frontal lobe. But, most of the time, TDCs from the other surfaces of frontal lobe mainly come from the MMA, which courses to the cerebral falx and anastomoses with the AFA 26. No statistical difference was noted in the TDCs to the other surfaces of the frontal lobe among different Suzuki stages, which might denote that theses TDCs form at the early stage of MMD.

Besides TDCs from around the frontal lobe, the posterior circulation also plays an important role in the compensatory mechanism of MMD 14, 27. Callosal circle is one of the most common collaterals from the posterior circulation. In this study, callosal circle from the PChA and PCA to the ACA accounted for 36% and 12% of the studied patients, respectively. Cranial base collaterals are also very important. They can provide blood supply to the ACA via the remnant PComA (24%), PCA-MCA pathway (28%), and moyamoya-like network (2%). Ventricular collaterals are uncommon and only accounted for 14% of the investigated patients in this study.

In MMD, enlarged PComA and AChA could provide more blood supply to the posterior circulation and thus increase collateral volume to the ACA territory 10, 28. In this study, enlarged PComA and AChA were noted in 54% and 17 % of the patients respectively. The moyamoya-like alteration could also involve the posterior circulation, which accounted for 6% of the patients in this study.

The perfusion of the frontal lobe is compensated by the aforementioned collaterals. There was no statistical difference in the vascularization state of the frontal lobe among the patients of different Suzuki stages. In this study, 80% of the patients showed good vascularization state in the frontal lobe, which indicated that, in the progression of MMD, the vascularization of the frontal lobe is relatively adequately compensated by the collateral circulation in patients with anterior cranial fossa TDCs.

A statistical analysis of the anterior cranial fossa TDCs with regard to the age groups and clinical manifestations was also performed. No statistical difference was noted between the child and adult groups. In addition, no statistical difference was noted between the ischemic and hemorrhagic groups either.

## Limitations

Blood supply to the frontal lobe was only evaluated through the vascularization state based on angiographic characteristics. No perfusion investigation (e.g. CT perfusion or magnetic resonance imaging perfusion) was analyzed. The angiography was obtained through large vessel angiogram and no selective angiography was performed. There was no long-term follow-up with catheter angiography, which is very important for evaluating the collateral development with the progression of MMD.

## Figures and Tables

**Table 1:** The Suzuki stage, frontal collaterals, and vascularization state of the patients

Suzuki stage	Number of hemispheres	Rete mirabile	AEA			PEA			Frontal TDCs from other sources	Vascularization (good)
II	5	1/5, 20%	I (4/5, 80%)	II (1/5, 20%)	III (0)	I (1/5, 20%)	II (0)	III (0)	1/5, 20%	4/5, 80%
III	46	28/46, 60.9%	I (24/46, 52.2%)	II (4/46, 8.7%)	III (14/46, 30.4%)	I (23/46, 50%)	II (3/46, 6.5%)	III (4/46, 8.7%)	19/46, 41.3%	44/46, 95.7%
IV	26	17/26, 65.4%	I (8/26, 30.8%)	II (3/26, 11.5%)	III (12/26, 46.2%)	I (12/26, 46.2%)	II (3/26, 11.5%)	III (5/26, 19.2%)	12/26, 46.2%	22/26, 84.6%
V	19	18/19, 94.7%	I (8/19, 42.1%)	II (1/19, 5.3%)	III (7/19, 36.8%)	I (7/19, 36.8%)	II (5/19, 26.3%)	III (6/19, 31.6%)	11/19, 57.9%	16/19, 84.2%
VI	4	3/4, 75%	I (0)	II (0)	III (3/4, 75%)	I (2/4, 50%)	II (1/4, 25%)	III (1/4, 25%)	2/4, 50%	3/4, 75%
Sum	100	67/100, 67%	I (44/100, 44%)	II (9/100, 9%)	III (36/100, 36%)	I (45/100, 45%)	II (12/100, 12%)	III (16/100, 16%)	45/100, 45%	89/100, 89%

**Abbreviations:** AEA, anterior ethmoid artery; PEA, posterior ethmoid artery; TDC, transdural collateral.

**Table 2:** Statistical analysis of the frontal collaterals and vascularization state with regard to Suzuki stages

Variables	Suzuki stage (Number of hemispheres)					Result
	Ⅱ (n = 5)	Ⅲ (n= 46)	Ⅳ (n = 26)	Ⅴ (n = 19)	Ⅵ (n = 4)	
Rete mirabile	1/5, 20%	28/46, 60.9%	17/26, 65.4%	18/19, 94.7%	3/4, 75%	*P<*0.05
AEA	5/5, 100%	42/46, 91.3%	23/26, 88.5%	16/19, 84.2%	3/4, 75%	*P>*0.05
PEA	1/5, 20%	30/46, 65.2%	20/26, 76.9%	18/19, 94.7%	4/4, 100%	*P<*0.05
Frontal TDCs from other sources	1/5, 20%	19/46, 41.3%	12/26, 46.2%	11/19, 57.9%	2/4, 50%	*P>*0.05
Vascularization (good)	4/5, 80%	44/46, 95.7%	22/26, 84.6%	16/19, 84.2%	3/4, 75%	*P>*0.05

**Abbreviations:** AEA, anterior ethmoid artery; PEA, posterior ethmoid artery; TDC, transdural collateral.

**Table 3:** Statistical analysis of the frontal collaterals with regard to clinical manifestations

Variables	Clinical manifestations		Result
	Ischemia (n = 48, 24 cases)	Hemorrhage (n = 42, 21 cases)	
AEA	45/48, 93.8%	39/42, 92.9%	*P>*0.05
PEA	35/48, 72.9%	30/42, 71.4%	*P>*0.05
Frontal TDCs from other sources	22/48, 45.8%	19/42, 45.2%	*P>*0.05

**Abbreviations:** AEA, anterior ethmoid artery; PEA, posterior ethmoid artery; TDC, transdural collateral.

**Table 4:** Statistical analysis of the frontal collaterals with regard to different age groups

Variables	Age groups		Result
	Child (< 18 years) (n = 10, 5 cases)	Adult (≥ 18years) (n = 90, 45 cases)	
AEA	8/10, 80%	81/90, 90%	*P>*0.05
PEA	8/10, 80%	65/90, 72.2%	*P>*0.05
Frontal TDCs from other sources	7/10, 70%	38/90, 42.2%	*P>*0.05

**Abbreviations:** AEA, anterior ethmoid artery; PEA, posterior ethmoid artery; TDC, transdural collateral.
